# Genome-Wide Dissection of Selection on microRNA Target Genes Involved in Rice Flower Development

**DOI:** 10.3390/plants13233281

**Published:** 2024-11-22

**Authors:** Fen Zhang, Li-Zhen Ling, Li-Zhi Gao

**Affiliations:** 1 Engineering Research Center for Selecting and Breeding New Tropical Crop Varieties, Ministry of Education, Tropical Biodiversity and Genomics Research Center, Hainan University, Haikou 570228, China; 2 Plant Germplasm and Genomics Center, Germplasm Bank of Wild Species in Southwest China, Kunming Institute of Botany, The Chinese Academy of Sciences, 132, Lanhei Road, Kunming 650204, China

**Keywords:** molecular evolution, sequence polymorphism, microRNA binding site, flower development, *Oryza sativa*

## Abstract

Although genome-wide studies have identified a number of candidate regions evolving under selection in domesticated animals and cultivated plants, few attempts have been made, from the point of a definite biological process, to assess sequence variation and characterize the regimes of the selection on miRNA-associated motifs. Here, we performed a genome-wide dissection of nucleotide variation and selection of miRNA targets associated with rice flower development. By sampling and resequencing 26 miRNA targets for globally diverse representative populations of Asian cultivated rice and wild relatives, we found that purifying selection has reduced genetic variation at the conserved miRNA binding sites on the whole, and highly conserved miRNA binding sequences were maintained in the studied rice populations. Conversely, non-neutral evolution of positive and/or artificial selection accelerates the elevated variations at nonconserved binding sites in a population-specific behavior which may have contributed to flower development-related phenotypic variation. Taken together, our results elucidate that miRNA targets involved in flower development are under distinctive selection regimes during rice evolution.

## 1. Introduction

MicroRNAs (miRNAs) are recently discovered short RNAs that were referred to as ‘dimmer switches’ of gene expression. They are initially transcribed as much longer single-stranded RNAs with imperfect hairpin structures, from which the mature miRNAs are excised by Dicer-like enzymes [1]. MiRNAs bind to their target mRNAs with a high degree of complementarity, and several computational approaches have been developed for the prediction of target genes at the genome level [2,3,4]. It was estimated that, in plants, at least 1% of protein-coding genes are regulated by miRNAs [2,5]. To date, 708 miRNAs from 334 miRNA families (miRBase, http://www.mirbase.org/ (accessed on 10 July 2021), Release 22.0) [6], including several natural antisense miRNAs (nat-miRNAs) and a mirtron, have been identified in rice [7,8]. Although the extent to which miRNAs contribute to phenotypic evolution is unclear, recent efforts have continuously dissected the important roles of miRNAs in regulating agronomically important traits in crops, such as ideal plant architecture in rice [9,10] and nodule formation in soybean [11]. Indeed, accumulating evidence has clearly demonstrated that miRNAs also play essential roles in a variety of biological processes including flower development [12]. For example, several conserved miRNAs, such as miR156, miR159, and miR172 [1,12,13], have been functionally characterized during the process of flower development. Overexpression or knockdown of these miRNAs disturbed metabolisms and consequently resulted in phenotype variation, including phase change, male sterility, early maturity, sex determination as well as floral organ identity [14,15,16].

Flower development is a prerequisite for the successful reproduction of flowering plants. In the past decades, enormous progress has been made in dissecting the molecular genetic underpinnings of flower development [17], but it is still too far away from outlining a full picture of the complicated regulatory network. Thankfully, the heart of such networks, such as important transcription factors (TFs) and their cis-regulatory elements, has a significant impact on the transcriptional regulatory cascades, therefore, much work has been devoted to understanding these key genes’ regulatory elements [18]. Mutations in these gene regulatory elements are more likely to affect certain kinds of phenotypic traits, and evolutionary selection operates more efficiently on these mutations.

MiRNAs may have co-evolved with their target genes when the targets experience functional divergence towards maintaining their regulatory functions [19]. It is possible that miRNAs, like protein-coding genes, also experienced selection during evolution. Thus, nucleotide mutations within sequences of target sites may influence miRNA regulation, and single-nucleotide polymorphisms (SNPs) naturally occurring in target sites can serve as ideal candidates for variation that may be of great significance in functional and evolutionary studies on various biological processes. Mutations occurring within such gene regulatory elements are intrinsically more likely to affect certain kinds of phenotypic traits, and therefore, natural selection operates more efficiently on these mutations [20]. Under the requirement of stringent recognition between a miRNA and its target site, naturally occurring SNPs may have significant functional implications for miRNA binding and posttranscriptional regulation. Selection constraints have been detected on miRNA binding sites based on genome-wide SNP data in human populations [21,22,23]. Great attention has also been paid to determining the signature of natural selection on miRNAs and their target sites based on large-scale surveys of miRNA polymorphisms in Arabidopsis, rice and soybean, respectively [16,24,25,26]. These results together indicate that miRNA target genes are always under strong selective constraints during plant genome evolution. Furthermore, it was found that minor mutations are positively selected, implying that those beneficial polymorphisms of miRNA target sites may exist in plants [16,24,25,27]. However, little is known thus far about the extent of polymorphisms at miRNAs and natural and/or artificial selection on miRNA-associated motifs involved in an important biological process, such as flower development in domesticated crops. Despite the importance of target transcription factors of conserved miRNAs in the regulation of plant flower development [1,12,13], whether these miRNAs serve as targets of natural and/or artificial selection and their potential contributions to phenotype evolution remain largely unclear during crop domestication.

Asian cultivated rice, *Oryza sativa*, is one of the most important cereal crops that was domesticated and experienced subsequent artificial selection during a long cultivation history of approximately 10,000 years [28,29,30]. The completion of genome sequencing of these two rice subspecies, *japonica* and *indica*, provides unprecedented opportunities to gain deep insights into the entire set of these regulatory elements involved in a development process [31,32]. In addition, different rice varieties present a wealth of diversity in reproductive characteristics. A large number of miRNAs have been identified from reproductive tissues/organs in rice, although their functions remain largely unknown [33,34,35,36]. All the aforementioned advantages make rice become an excellent model to comprehensively reveal patterns of nucleotide polymorphisms and explore evolutionary mechanisms of miRNA modules relevant to flower development in flowering plants.

To detect signatures of selection on miRNA targets involved in rice flower development, we evaluated levels and patterns of their nucleotide polymorphisms by resequencing 26 miRNA target sites for a panel of 74 rice accessions with a global range of geographical and genetic origins. They include the three major subsets of *indica*, temperate *japonica* and tropical *japonica* of *O. sativa* and its two presumed wild progenitors, *O. rufipogon* and *O. nivara*. We found that almost no variation occurred at conserved binding sites but high polymorphisms presented within nonconserved binding sites involved in rice flower development. Our results suggest that conserved binding sites are under strong purifying selection. Nonetheless, nonconserved binding sites are subject to non-neutral evolution by serving as putative targets of selection during rice domestication, possibly contributing to phenotypic variations in rice flower development.

## 2. Results

### 2.1. Prediction of miRNA Targets Involved in Rice Flower Development

In this study, computational prediction has identified a total of 53 high-probability targets for 22 miRNA families (Supplementary Table S2). We then incorporated experimental data to estimate the accuracy of predicted miRNA targets, and found that up to 33 were experimentally validated in previously published references [7,25,33,34,36,37,38,39,40,41,42,43,44] and documented in the updated miRBase database (Release 22.0, http://microrna.sanger.ac.uk (accessed on 1 July 2021)) (Supplementary Table S2). While the remainder needs further experimental confirmation, their matching miRNAs were indeed expressed in rice reproductive organs [8,44,45,46]. Overall, our results suggest that the prediction of miRNA targets was sufficiently accurate. Such data offer evidence that the majority, if not all, of these flower-related genes may be regulated by their miRNAs. However, it is likely that some real non-canonical miRNA targets may have been overlooked in pursuit of high accuracy and reliability merely by means of computational predictions.

Among 53 predicted target genes that may be involved in rice flower development, the majority were transcription factors, such as *MYB*, *MADS-box*, and *AP2/EREBP*, while others were mostly involved in signal transduction pathways and components regulated feedback circuit of the miRNA pathways (Supplementary Table S2). It is obvious that the physical locations of predicted miRNAs binding sites were not symmetrical. Of these binding sites, 42 were located in coding regions (CDS), 8 in 3′ untranslated regions (UTRs), two in 5′ UTRs, and one in CDS-3′ UTR of target genes (Supplementary Table S2). These observations appeared in agreement with the previously reported findings that most binding sites are predominantly located in coding regions of target genes [2]. Likewise, it indicates that, with the exception of coding regions, miRNAs also mediate regulation via binding to UTRs in the process of plant flower development.

### 2.2. Naturally Occurring Variation Within miRNA Binding Sites

We totally sampled 26 predicted miRNA target sites related to flower development to characterize naturally occurring variation within miRNA binding sites in the cultivated rice and wild rice populations. Among these binding sites resequenced in the 26 *Oryza* accessions, we found that a total of two target sequences (*LOC_Os01g18850*, *LOC_Os09g23620*) were variable with the number of segregating sites (S) not equal to zero (Supplementary Table S2). It is worthwhile pointing out that nucleotide variation of the two binding sites was common in both cultivated rice varieties and wild rice populations with the exception of *LOC_Os01g18850* and *LOC_Os09g23620*. To verify whether the observed variation in the two targets above mentioned could be affected by a small sample size, we repeatedly increased the number of samples and detected single nucleotide substitution from an individual of *O. rufipogon* (Supplementary Table S2). Finally, we were able to confirm that there were a total of two binding sites, which naturally occur reliable variation in the whole panel of the examined rice accessions.

The two variable genes were found to be targeted by one rice-specific miRNA family, miR818 and one conserved miRNA family miR162 (Supplementary Table S2 and Figure S1). In contrast, non-variable binding sequences are mainly targeted by evolutionally conserved miRNAs across the majority of plant lineages, including mosses, gymnosperms, monocots and eudicots (Supplementary Figure S1). The results imply that conserved and nonconserved miRNAs may differently behave in their regulatory functions during rice flower development.

### 2.3. Reduced Polymorphisms in and Purifying Selection on Conserved miRNA Binding Sites

To gain insights into the evolution of miRNA target genes involved in rice flower development, we classified the whole set of binding sites into two subsets: first, sites that are targeted by conserved miRNAs, called conserved miRNA binding sites; and second, sites that are targeted by rice-specific miRNAs, namely nonconserved miRNAs or rice-specific binding sites. In this study, there were 24 conserved miRNA binding sites, while the other two could be classified into rice-specific binding sites (Supplementary Table S2 and Figure S1). For both two subsets, we observed significant differences at levels of the global mean nucleotide diversity. The estimated mean *π* at the conserved miRNA binding sites was two times lower than that at the nonconserved miRNA binding sites in rice cultivars and wild ancestral populations (*π* = 2.76 and *π* = 9.83 for rice cultivars, Wilcoxon rank sum test: *p* = 0.048; *π* = 4.82 and *π* = 11.43 for wild ancestral populations, Wilcoxon rank sum test: *p* = 0.026) (Table 1). When compared to the background level of polymorphisms previously estimated by using 111 STS of gene fragments across the genome [47], polymorphisms in conserved miRNA target sites were also lower than that in the 111 STS for both the cultivated rice and wild ancestral populations (Table 1). These results indicate that conserved miRNA binding sites present rather lowered nucleotide diversity in both cultivated rice and wild ancestral populations.

**Figure 1:**
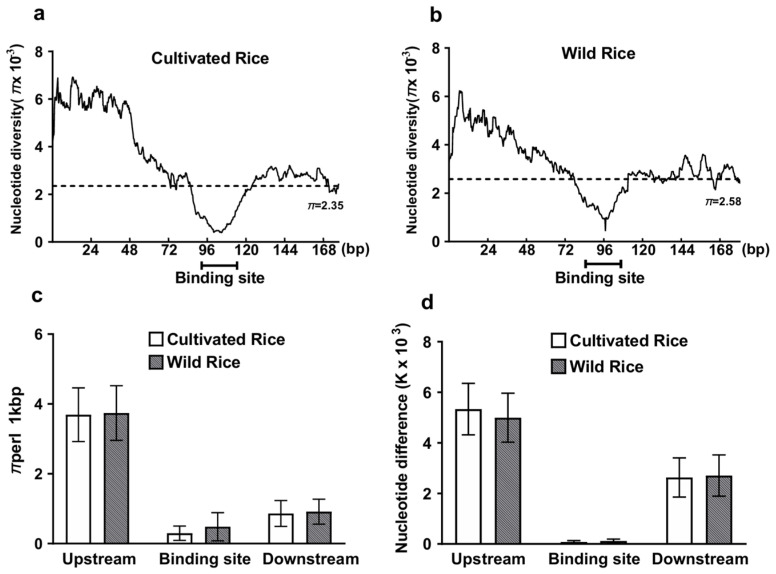
Sliding-window analysis of nucleotide diversity in conserved miRNA binding sites. (**a**) in cultivated rice; (**b**) in wild ancestral populations. The window is 21 nt across (corresponding to the mean size of miRNA target sites), with a size step of 1 nt. The dashed line represents distribution pattern of mean nucleotide diversity of random sequences, as shuffled by the resampling procedure (see Materials and Methods). Mean levels of polymorphisms (**c**) and nucleotide divergence (**d**) are also given at conserved miRNA target sites and flanking regions.

Lowered polymorphisms could equally be explained by purifying selection and nonselective evolutionary processes, such as genomic regions with extremely low mutation rates known as mutation “cold spots” [23]. To clarify whether the reduced diversity in these conserved miRNA target sites is due to purifying selection or lowered regional mutation rate, we performed a sliding-window analysis (window size equals mean size of binding sites, 21) of nucleotide diversity *π* over different genomic regions. Under the mutation “cold spots” hypothesis, a random fluctuation of *π* was expected over the sequenced regions. Whereas, purifying selection should reduce diversity exclusively operating on functional domains within miRNA binding sites. Consistent with the hypothesis of purifying selection, the sliding window graph showed that conserved miRNA binding sites were the most highly constrained domains and displayed the lowest polymorphisms (Figure 1a,b). Furthermore, the distribution of genetic diversity within randomly shuffled sequences is different from true binding sequences, suggesting that genetic diversity in conserved binding sites is not distributed at random and these regions are indeed under purifying selection.

To further look for the evidence that purifying selection has resulted in a reduced diversity in these conserved miRNA target sites, we estimated and compared levels of nucleotide diversity (*π*) between conserved miRNA binding sites and their flanking sequences. As shown in Figure 1c, the conserved miRNA binding sites exhibited obviously lower nucleotide diversity than their flanking regions for both rice cultivars and wild ancestral populations. In the cultivated rice, we failed to detect any segregating sites in the conserved miRNA binding sites (Figure 1c and Supplementary Table S2), while relatively higher nucleotide diversity was found with *π* = 3.69 and *π* = 0.87 at the 5′ and 3′ flanking regions, respectively (Wilcoxon rank sum test, *p* < 0.042). A similar observation also appeared in wild rice populations (Figure 1c). The results again suggest that these conserved miRNA binding sites are subject to strong selective constraints in both cultivated rice and wild rice populations. The results seem in agreement with another study on rice, which previously reported that purifying selection was the predominant evolutionary force acting on miRNA sequences [16].

To detect more distant evolutionary events than the assessment of polymorphisms and potentially resolve stronger selective effects, we then estimated and compared levels of divergence (k) between conserved miRNA binding sites and their flanking regions using the African wild rice *O. barthii* as outgroup. Nucleotide divergence at the conserved miRNA binding sites was k = 0.0001/0.0002, significantly lower than that at either upstream (k = 0.0053/0.0049) or downstream (k = 0.0026/0.0027) sites (Figure 1d) in both cultivated rice and wild rice populations, respectively. Collectively, these results indicate that strong purifying selection may have acted on the conserved miRNA binding sites in the two studied rice species.

To develop a broader model of polymorphisms at conserved miRNAs of rice flower development genes, we estimated levels of nucleotide diversity (*π*) between mature miRNA sequences and their flanking sequences by retrieving miRNAs associated with flower development reported previously [16] (Supplementary Table S5). By analyzing 31 miRNAs (839 sequences) in cultivated rice and 9 miRNAs (90 sequences) in wild rice, we found that the 5′ and 3′ flanking regions (*π* = 0.0043 and *π* = 0.0040, respectively) exhibited relatively higher nucleotide diversity than mature miRNA sequences (*π* = 0.0004) in cultivated rice. The SNP density in cultivated rice was found to be 0.0061, 0.0041 and 0.0002 SNPs/bp in the 5′, 3′ flanking regions and binding sequences, respectively. Comparisons of nucleotide diversity and SNP density revealed a similar pattern between mature miRNA sequences and their flanking sequences in wild rice (Supplementary Table S5). These results further strengthen that a stronger purifying selection predominantly acts on the conserved miRNA binding sites than surrounding sequence regions during the co-evolution of miRNAs and their targets of floral development genes.

### 2.4. Elevated Variation and the Signature of Natural Selection and/or Artificial Selection in the Two Rice-Specific miRNA Binding Sites

**Figure 2:**
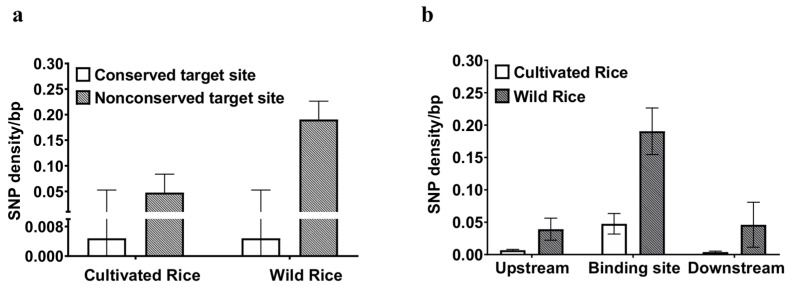
Comparisons of SNP densities in conserved binding sites and nonconserved binding sites (**a**), and rice-specific miRNA binding sites and their flanking regions (**b**) between cultivated rice and wild ancestral populations. Error bars represent the standard errors of the mean values.

For the two variable rice-specific miRNA binding sites, the sample size was increased to 74 varieties (Supplementary Table S1) and a total of 9 SNPs (including indels) were identified by genome resequencing (Table 2). We first compared levels of nucleotide diversity between nonconserved binding sequences and 111 STS [47]. Levels of nucleotide polymorphisms within these sequences were significantly higher than those background levels for 111 STS in cultivated rice (*π* = 9.83 and 3.20, respectively, Wilcoxon rank sum test, *p* < 0.05) (Table 1). A similar pattern was observed in wild rice populations (*π* = 11.43 and 5.19, respectively, Wilcoxon rank sum test, *p* < 0.05) (Table 1). The SNP density in rice-specific miRNA binding sites was 0.047 and 0.19 SNPs/bp in cultivated rice and wild ancestral populations, respectively. They were significantly higher than those in conserved miRNA binding sites (0.00048 and 0.00048 SNPs/bp for cultivated rice and wild ancestral populations; χ^2^, *p* < 0.0001 for both) (Figure 2a). We also compared the SNP density between the rice-specific binding sites and their upstream or downstream flanking regions. The rice-specific miRNA binding sites unexpectedly showed a higher SNP density than their flanking regions in both cultivated rice and wild ancestral populations (Figure 2b). Such a finding demonstrates that an accelerated evolution may have occurred in the rice-specific miRNA binding sites.

Since SNP density is sensitive to mutation rate heterogeneity, we further analyzed the derived allele frequency (DAF), which does not rely on the variation of mutation rates [48]. In comparison to neutral expectation, an excess of high-frequency derived variants is the salient signature of positive selection [49]. In this study, the ancestral allele states were inferred using *O. barthii* as outgroup. Of these derived alleles, only one was shared among all the populations at each locus, such as the allele 4 at *LOC_Os01g18850*, and allele 5 at *LOC_Os09g23620* (Table 2). The other alleles were not present in all rice populations but in one or two populations, and so forth (Table 2). We observed that allele frequencies substantially differed across populations (Chi-square test (χ^2^ test), *p* < 0.0001) (Table 2). For the shared derived alleles, they showed rather high frequencies (i.e., above 80%) within each *O. sativa* population and were almost fixed across the studied cultivated rice samples (Table 2). By contrast, the frequencies of the remaining alleles were relatively low in cultivated rice. Unlike cultivated rice, wild rice populations showed high or low frequencies of shared derived alleles as well as the remaining alleles (Table 2). However, the high-frequency derived alleles in cultivated rice also existed in wild ancestral populations. Aside from positive selection which acts on both cultivated rice and wild ancestral populations and thereafter results in the elevated variation, it is likely that genetic bottleneck and artificial selection could determine the observed outcomes during the domestication of cultivated rice. To test the possibility, we compared the reduction of genetic diversity between candidate genes in this study and 111 STS distributed across the whole *O. sativa* genome [47]. The level of nucleotide diversity (*π*) at rice-specific targets was 0.004 in *O. sativa*, while the estimated *π* value for the *O. rufipogon* population was 0.009, representing a 56% reduction in molecular variation. The genome-wide reduction was 36% on average [47], indicating that the bottleneck could not solely account for the lowered diversity in rice cultivars. Together, the excess of high-frequency derived mutations in all rice populations and the exclusion of bottleneck effect in rice cultivars provide the evidences of positive selection and/or artificial selection on rice-specific miRNA binding sites.

To examine whether these derived alleles deviate from neutral expectation, we calculated the value of Fay and Wu’s H, the most commonly used test for highly derived alleles [50]. The H statistic suggests that there was a strong departure from neutrality, which is consistent with artificial selection within different populations of cultivated rice for each locus (*p* < 0.05), particularly in the *japonica* varieties (Table 3). The wild ancestral populations exhibited much more polymorphic sites than rice cultivars (Figure 2b, Table 2 and Supplementary Table S1), and hence should be powerful to detect the departure from neutrality. Yet, we failed to succeed in observing such a departure as strong as expected (*p* > 0.05) (Table 3). To further investigate positive selection in these small RNA loci, in addition to the Fay and Wu’s H test, we performed the MLHKA test [51] for each nonconserved miRNA binding site by using 10 neutrally evolving genes that are believed to have not been affected by selection [52]. The test is able to estimate the degree of nucleotide diversity reduced by positive selection, and the rejection of the multi-locus MLHKA test that measures the difference in diversity within species relative to divergence between species is often considered as the evidence of a positive selection. As shown in Table 3, the MLHKA test was not significant for two wild species but significant merely in the *japonica* cultivars at rice-specific miRNA binding sites. Accordingly, the signals were not shared between cultivated rice and wild ancestral populations, these are indicative of cultivated rice-specific selection events, especially in the *japonica* subspecies. Taken as a whole, our findings demonstrate that a rapid and non-neutral evolution has operated on these rice-specific miRNA binding sites through positive selection and/or artificial selection.

### 2.5. Variants Located Within Rice-Specific miRNA Binding Sites May Affect miRNA–Target Interactions

To evaluate possible impacts of variants, including both SNPs and indels, on the coevolution of miRNAs and their targets, we first calculated MFEs of miRNA/target duplex formation based on each observed haplotype of target sequences (Table 4). The thermodynamic data showed that these variants with more mismatches were likely to affect the stability of the duplex, a characteristic of smaller negative MFEs (Table 4). After the elimination of the influence of target sequences in length, the normalized MFEs hold consistent results in this study (Table 4). In addition, we observed that the mismatches occurring at different positions of target sequences with the same number of mismatches led to different MFEs. Taken together, these observations of base pairing and other attributes of miRNA–target interactions suggest that most variants might be helpful to make differences in target gene expression by destabilizing the duplex.

We next assessed the influence of variants on the gain and loss of miRNA–target interaction sites. To conveniently identify these events, we divided all mutants located in target sites into three categories: first, occurring within mismatch positions; second, creating a nondisruptive mismatch from a match; and finally, creating a disruptive mismatch from a match. The first class of mutants could not affect the gain or loss of miRNA targets, whereas the latter two classes of mutants provided additional opportunities to create or disrupt a target site. For example, the presence of new alternative alleles in haplotype 2 or haplotype 4 of *LOC_Os01g18850* turned out to be novel binding sites. Furthermore, more than four mismatched haplotypes presented in *LOC_Os09g23620*, respectively, did not serve as targets by using prediction criteria in the present study. Our analysis demonstrates that these variants may have significantly contributed to target gene expression as well as miRNA–target gene modules that gain or lose their functions during evolution.

To understand which mutation may affect the gain and loss of the miRNA target sites, we selected 12 rice strains to sequence genomic regions of the rice-specific (miR818) putative pre-miRNAs and summarized genetic variation of these miRNA sequences by presenting the putative miRNA: target pairing between miRNAs and their predicted targets (as given in Supplementary Table S6). Our results showed that not all haplotypes (Table 4) were matched by the corresponding mutation miRNAs. However, a perfect match miRNA–target base pair was observed (as shown in Supplementary Figure S2). This indicates that the rice-specific miRNAs were newly generated so that the putative miRNA: target pairing has not been stably formed.

## 3. Discussion

A fundamental question in biology is how selection affects the evolution of developmental processes by acting on key regulators such as miRNAs and their target sites. In recent decades, levels of variation at miRNAs and their target sites have been genome-wide surveyed in *Arabidopsis* and rice [16,24,25]. Most of these sites exhibit lowered polymorphisms and are subject to strong purifying selection, whereas a small number of them have undergone positive or balanced selection. Such results have provided us clues for whether distinctive selection mechanisms govern the entire layer of miRNA regulation modules. In this study, we genome-wide assessed polymorphisms of the putative miRNA targets involved in flower development by resequencing a globally representative sample of cultivated rice and wild ancestral populations. The efforts allow us to examine how such a class of regulatory elements has been targeted by selection during the process of rice flower development.

Our results suggest that miRNA target sites involved in rice flower development evolve under different selective forces. For the conserved miRNA targets, purifying selection serves as a primary evolutionary force and thus plays an important role in the conservation of rice flower development from one to another species. Levels of sequence polymorphism and divergence in these binding sites appeared lower than those in their flanking regions. The results seem somewhat in agreement with other studies in rice [16] and soybean [26]. They reported that most of miRNA family members corresponding to candidate target genes in the present study have undergone strong purifying selection. Numerous studies have shown that floral organ identity factors have been fairly conserved in their functions in rice, *Arabidopsis*, and other higher eudicotyledonous flowering plants [53,54]. Thus, our findings, together with strong selective constraints depicted in the studied target sites, corroborate that the role of conserved miRNA–target interactions is essential in rice flower development. The global signature of purifying selection was represented within all studied rice populations, suggesting that these miRNAs regulatory modules are subject to being conserved during flower development in rice species. The striking parallels of conserved miRNA modules have been documented across the vast phylogenetic distances. MiR156/172-target interactions during flower development, for example, have been similarly preserved between *A. thaliana* and maize [55,56,57].

We detected a number of accumulated mutations in rice-specific miRNA targets, which is a likely indicator of advantageous mutations undergoing positive selection. Elevated SNP density, high derived allele frequency, and sequence-based neutrality tests together showed robust signatures of positive selection in cultivated rice and wild ancestral populations, particularly population-specific positive selection in cultivated rice after precluding the noise caused by domesticated bottleneck. As a rule, two genes which interact in the signaling network tend to coevolve and be targeted by the same selection force during evolution. Similar results were formerly found that human-specific miRNAs experience robust signatures of positive selection [58]. Note that miRNAs and associated regulatory elements involved in the same regulatory pathway may tend to be targeted by the same selection forces during evolution. Nevertheless, little has been known about detailed functions of the two nonconserved miRNA target genes of *LOC_Os01g18850* and *LOC_Os09g23620* in this study. Therefore, how these rice-specific miRNAs were driven and conserved by positive selection across diverse *Oryza* species in rice flower development deserves further functional studies.

This study demonstrates that the studied nonconserved miRNA targets involved in flower development may have experienced artificial selection in cultivated rice, as a significantly positive selection signal was detected in the cultivated varieties but not in the wild ancestral populations. Recent genome-wide characterization of miRNA genes in rice also suggests that a handful of genes involved in the same regulatory pathways could be targeted by artificial selection [16]. In this study, the *japonica* subgroup was found to have experienced stronger positive selection than the *indica* subgroup. Subspecific positive selection has strongly accelerated adaptive evolution of miRNA targets involved in flower development, which could enhance rapid functional divergence and promote their fitness in the changeable environments between *indica* and *japonica*. Our results thus suggest that miRNAs and possibly more other small RNA loci involved in flower development endeavor to deserve deep insights into the molecular basis of rice domestication and improvement. Further experiments are needed to reveal the consequences of positive selection, such as phenotypic changes, along major pathways in the decision to floral development from one to another.

The comparison revealed that evolutionary changes actively occurred in nonconserved miRNA target sequences, thus possibly making more contributions to species-specific characteristics resulting in the diversified and evolved functions of flower development. Our analyses show that the mutations disrupt ligand binding between nonconserved miRNA and its binding sites and destabilize structures of miRNA/target duplexes in rice. These destabilizing effects may be caused by altering the number and location of mismatches. Considerable studies suggest that altered ligand binding is responsible for regulatory diversity and subsequent variable phenotypes [59]. However, whether these variations at rice-specific miRNA target sites might have changed the gene regulation system and confer phenotype diversity remain unanswered. We also observed that the gain and loss of miRNA target sites could serve as a major source for miRNA functional diversity. These results imply that positive selection opens the possibility of elevated variations at nonconserved miRNA target sites and drives the diversification of miRNA regulatory modules during the process of flower development in rice species. Ehrenreich and Purugganan [24] formerly proved that variants in miRNA genes, which destabilize the secondary structure of pre-miRNA under different temperatures, could also be used as raw materials for differential functions of miRNA alleles in *A. thaliana*. Nonetheless, whether it similarly occurs in nonconserved rice miRNA genes remains unresolved before further studies.

## 4. Materials and Methods

### 4.1. Sample Collection and DNA Isolation

A panel of 74 diverse accessions of *O. sativa* and its two presumed wild progenitors, *O. rufipogon* and *O. nivara*, was sampled to span a wide range of their geographical origins by using one *O. barthii* accession originally collected from Africa as an outgroup, on behalf of an abundant genetic diversity within these species. Here, the three major groups of *O. sativa* were represented by 50 varieties, including 16 of *indica*, 17 of temperate *japonica* and 17 of tropical *japonica*. The panel also comprised 19 of *O. rufipogon* and 5 of *O. nivara*, totaling 24 wild accessions. In this study, one *O. barthii* accession originally collected from Africa was used as an outgroup. This wild species is closely related to *O. sativa* and its wild progenitors [60,61,62] but away from the introgression one another due to geographical isolation. All the studied accessions with their scientific names and geographic origins were given in Supplementary Table S1. The majority of these rice germplasms were kindly provided by the International Rice Research Institute (Manila, Philippines), while others were collected from natural populations of *O. rufipogon* in China. Seeds were germinated on filter paper, and then the seedlings were transplanted into plots with soil in the greenhouse. Total DNA was isolated from fresh leaf tissues using the CTAB method [63].

### 4.2. Prediction of miRNA Target Genes Involved in Flower Development

Mature rice miRNA sequences were downloaded from the miRBase database (Release 22.0, http://microrna.sanger.ac.uk (accessed on 3 July 2021)) [6], followed by removing redundant miRNA sequences. For the prediction of miRNA targets, we employed psRNATarget software (https://www.zhaolab.org/psRNATarget/ (accessed on 6 July 2021)) [4] to search against cDNA database of the RGAP rice genome (version 7.0, http://rice.plantbiology.msu.edu/ (accessed on 6 July 2021)). To obtain more putative targets, we selected a relatively loose threshold with maximum energy to unpair the target site (UPE) as 50.0 (range 0–100) and default options otherwise. Putative functions of the predicted miRNA targets were then annotated based on Plant GOSlim Ontologies (http://rice.plantbiology.msu.edu/downloads_gad.shtml (accessed on 8 July 2021)). The genes were collected with GO terms of biological processes associated with flower development. In order to identify as many flower development-related genes as possible, we first made queries against other databases, including massively parallel signature sequencing (MPSS) and The National Center for Biotechnology Information (NCBI), by searching keywords of ‘flower’ and ‘floral’. Additional confirmation was made by querying against the expression database (http://rice.plantbiology.msu.edu/expression.shtml (accessed on 8 July 2021)) including OSN_A, OSN_AD, and OSN_AE by setting the value >0. Together, 80 target genes that have binding sites for miRNAs in 58 miRNA families were totally collected. Considering the major downside of in silico approaches may result in the occurrence of false hits, we used the second filter, empirical parameters, to minimize false hits [64]. G:U pairs were treated as mismatches, and the cut-off of score 3 was taken. The applied parameters were much more stringent as follows: no mismatch at positions 10 and 11; no more than one mismatch at positions 2–12; no more than two consecutive mismatches downstream of position 13; and the total number of mismatches fewer than 5. Finally, a total of 53 high-probability targets for 22 miRNA families were obtained and given in Supplementary Table S2.

### 4.3. PCR Amplification and DNA Resequencing

To amplify predicted miRNA binding targets and correlative pre-miRNA sequences, we designed primers in flanking sequences based on the *Nipponbare* reference genome using software Primer Premier 5 (http://www.premierbiosoft.com/ (accessed on 8 July 2017)). All primer pairs used for PCRs were conducted blast searches against the rice genome sequence to ensure their specificity so that each targeted genomic region and pre-miRNA sequence could be uniquely amplified (Supplementary Tables S3 and S4). PCR reactions were performed as previously described [64]. The amplified targets and pre-miRNA sequences of *O. sativa* and *O. nivara* were directly sequenced with ABI 3730 sequencing machine at BGI (Beijing Genome Institute, Shenzhen, China), while the PCR fragments of *O. rufipogon* were cloned into pMD18-T vector (Takara) and at least three clones were sequenced using the forward or reverse primer. Finally, a total of 26 target sequences for 75 rice strains and 7 pre-miRNA sequences for 12 rice strains were successfully amplified. The target expected mean size was 716 bp, corresponding to a total of 4.17 kb of genomic regions (Supplementary Table S3), while the total length of sequenced pre-miRNAs was approximately 47 kb (Supplementary Table S4). Sequences obtained in this study were deposited in the GenBank database under accession numbers JN192502-JN193387.

### 4.4. Data Analyses and Neutrality Tests

Sequences were initially aligned and edited by using SeqMan (DNASTAR, https://www.dnastar.com/ (accessed on 8 August 2022)) and BioEdit version 7.0.9 [65]. Multiple sequence alignments were performed using CLUSTALX V1.83 [66] and then refined manually. The haplotypes were assigned based on Markov Chain Monte Carlo (MCMC) algorithm by the program PHASE [67] in DnaSP V5.10.01 [68]. Unless otherwise specified, insertion/deletions (indels) were not included in the analysis. Sequences of 26 miRNA binding sites were directly extracted from *Nipponbare* (temperate *japonica*) [32] genome sequences and further included in subsequent data analyses. For each locus and taxon, we calculated the number of segregating sites (S), nucleotide diversity per site (*π*), and nucleotide substitution rates (K) by using DnaSP version 6.12.03 [69]. SNP density per region was estimated as the ratio between the number of segregating sites observed and the sequence length of the region. After excluding individuals with more than three indels at binding sites, allele frequencies for each SNP at binding sites were manually determined. For the entire sequence fragment containing each miRNA, the tests of neutral theory were estimated using two different methods: Fay and Wu’s H test [49,50] and maximum likelihood multilocus Hudson-Kreitman-Aguade (MLHKA) test [51]. For the MLHKA test, a set of ten neutral genes was employed as reference loci [52]. Sequences of *O. barthii* were used as an outgroup to identify ancestral alleles for each SNP before neutral tests were performed. Genome-wide background diversity, as reported previously [47], was estimated by using a total of 111 sequence-tagged sites (STS) of gene fragments randomly distributed throughout the whole rice genome. Wilcoxon rank sum and χ^2^ tests were conducted through SPSS v15 (SPSS Inc., Chicago, IL, USA).

### 4.5. Sequence Resampling

To assess whether nucleotide diversity at these targets of conserved miRNAs with respect to flanking regions possesses nonrandom distribution, we compared the mean of nucleotide diversity π obtained from 21 nt sliding windows (of step 1) to that of the random sequences. In this study, the 21 nucleotides correspond to the mean size of all predicted target sites. Sequence resampling was conducted as follows: 1000 shuffled sequences were first generated following target-specific dinucleotide frequencies [52] for each *Nipponbare* target sequence with ambiguous codes at polymorphic sites of each locus. Each shuffled sequence was then applied to all population sequences to ensure uniform permutation, and simultaneously accompanied by restoring of original SNP allele within the sequence. Next, *π* values were calculated according to the calibration of the above-mentioned sliding window for these randomly shuffled population fragments. We finally obtained the distribution of the mean π from these random population sequences.

### 4.6. Computation of Hybridization Energy of miRNA/Target Duplex

The specificity of the miRNA: mRNA interactions derives from the stability of intermolecular base pairing. To evaluate the effect of naturally occurring variations within the binding sites on the stability of miRNA–target heterodimer, we estimated the minimum free energy (MFE) using the program RNAcofold from the ViennaRNA package (http://rna.tbi.univie.ac.at/ (accessed on 8 August 2022)) [70]. To eliminate the influence of sequence length of binding sites and miRNAs, we normalized MFEs according to Rehmsmeier’s method [71]. The negative normalized energy (en) is defined as follows:

$$e_{n}=-\frac{e}{\log\left(mn\right)}$$

where, e is the minimum free energy; m and n are the length of the target sequence searched and the mature miRNA, respectively.

## 5. Conclusions

We investigated sequence variation at miRNA target genes and found that two classes of miRNA targets involved in flower development are under different selection regimes during rice evolution. The signature of purifying selection globally restraining the mutations of conserved miRNA binding sequences indicates an essential role of these molecules in modulating the gene-regulatory network during rice flower development. By contrast, positive selection shapes the variation of nonconserved miRNA targets, suggesting that these regulatory factors could confer the flower-related attributes on the rice species. Moreover, the finding that significant positive selection was specifically detected in cultivated rice and particularly in the *japonica* subgroup demonstrates an important role of artificial selection on small RNA loci in rice domestication and improvement. Experimental assays will determine potential functional effects of these variants within nonconserved miRNA target sites. Despite their deleterious, neutral, or advantageous fitness effects, these nonconserved miRNA variants that may be indeed under positive selection and thus affect expression will certainly shed light on mechanisms for the regulation and variability of flower-related phenotypes among the different rice populations.

## Figures and Tables

**Table 1:** Average diversity measures in rice cultivars and wild populations.

Statistics	Populations	Background Diversity ^b^	Conserved		Nonconserved	
			Target Sites	Average Size (bp)	Target Sites	Average Size (bp)
*π* ^a^ (per kb)	Cultivated rice	3.20	2.76	572	9.83	635
	Wild rice	5.19	4.82	570	11.43	646

^a^ Average synonymous nucleotide diversity under different categories; ^b^ Background diversity: background diversity was estimated by using a total of 111 sequence-tagged site (STS) gene fragments randomly distributed throughout the rice genome [47].

**Table 2:** Derived allele frequency analyses in rice-specific binding sites within each population of *O. sativa*, *O. nivara* and *O. rufipogon*.

NO	Target Gene	Allele ^a^	IND ^b^	TEM ^b^	TRO ^b^	JAP ^b^	ON ^b^	OR ^b^
1	*LOC_Os01g18850*	C	0	0	0	0	0	0.05
2		G	0	0	0	0	0	0.11
3		T	0.06	0.17	0.35	0.26	0	0.79
4		G	0.82	0.94	0.88	0.91	0.60	0.37
5	*LOC_Os09g23620*	T	1	1	0.94	0.97	0.20	0.53
6		G	0	0	0.06	0.03	0	0.05
7		T	0	0	0.06	0.03	0	0.05
8		T	0	0	0.06	0.03	0.80	0.58
9		T	0	0	0	0	0.20	0.05

^a^ the ancestral states of alleles were inferred by using *O. barthii* as outgroup; ^b^ IND-*indica*; TEM-temperate *japonica*; TRO-tropical *japonica*; JAP-*japonica* in total; ON-*O. nivara*; OR-*O. rufipogon*.

**Table 3:** Tests for selection and hitchhiking.

Taxon	*LOC_Os01g18850*			*LOC_Os09g23620*		
	H ^a^	MLHKA ^b^	Average Size (bp)	H ^a^	MLHKA ^b^	Average Size (bp)
*indica*	0.005 **	0.760	473	0.735	0.820	730
*japonica*	0.281	0.032 *	474	0.006 *	0.022 *	730
Temperate *japonica*	0.016 *	0.034 *	474	0.166	0.030 *	730
Tropical *japonica*	0.437	0.032 *	474	0.014 *	0.020 *	729
*O. nivara*	0.134	0.850	474	0.691	0.991	723
*O. rufipogon*	0.182	0.940	473	0.753	0.991	729

^a^ The *p* value of Fay and Wu’s H test; ^b^ The probability of chi square distribution of multilocus MLHKA test. * *p* < 0.05; ** *p* < 0.01.

**Table 4:** The haplotypes of target regions and the hybridization energy of miRNA–mRNA heterodimer ^a^.

Target Gene	Haplotypes	MFE	Normalized MFE
*LOC_Os01g18850*	(1) CCGUCCCAUAAUAUAAGGGAUU	−36.85	−13.76
	(2) C**U**GUCCCAUAAUAUAAGGGAUU	−34.68	−12.92
	(3) CCGUCCCAUAAUAUAAGG-AUU	−31.04	−11.56
	(4) CC**A**UCCCAUAAUAUAAGGGAUU	−31.18	−11.61
	(5) CCGUC**U**CAUAAUAUAAGG**A**AUU	−27.97	−10.42
*LOC_Os09g23620*	(1) CCGUCCCAUAAUAUAAG**A**GAUU	−29.96	−11.16
	(2) C**G**GUC**U**CAUAAUAUAAG**A**GAUU	−22.99	−8.56
	(3) C**G**GUC**U**CAUAAUAUAAG**A**GAU**C**	−22.42	−8.35
	(4) **UG**GUC**U**CAUAAUA**C**AAG**A**GAUU	−18.95	−7.06

^a^ The haplotypes were phased in target regions, while the mismatches or gap positions including the polymorphic sites were in bold.

## Data Availability

All data generated or analyzed during this study are included in this published article and its Supplementary Materials.

## Supplementary Materials

The following supporting information can be downloaded at: https://www.mdpi.com/article/10.3390/plants13233281/s1, Figure S1: The distribution of miRNAs in plants; Figure S2: Target genes *LOC_Os01g18850*/*LOC_Os09g23620* targeted by miR818; Table S1: *Oryza* plant materials used in this study; Table S2: List of predicted target genes involved in flower development in rice; Table S3: Primer sequences and expected product size for PCR amplifications; Table S4: Primer pairs for amplification of pre-miRNAs; Table S5: Average diversity measures of miRNAs involved in flower development in rice cultivars and wild populations; Table S6: The genetic variation of the two rice-specific miRNA sequences. Ref. [72] is cited in the Supplementary Materials.
