# Supplementary material for: Genome-Wide Dissection of Selection on microRNA Target Genes Involved in Rice Flower Development
Source: Plants (Basel). 2024 Nov 22;13(23):3281. doi: 10.3390/plants13233281 (PMC11644493; doi:10.3390/plants13233281)
Supplement: Supplementary file 1 [file plants-13-03281-s001.zip › plants-3236798-supplementary.pdf]

Supplementary Information

Genome-Wide Dissection of Selection on MicroRNA Target Genes Involved in Rice Flower Development

Fen Zhang[1†], Li-Zhen Ling[3†], Li-Zhi Gao[1,2*]

The following Supporting Information is available for this article:

Figure S1-S2

Table S1-S6

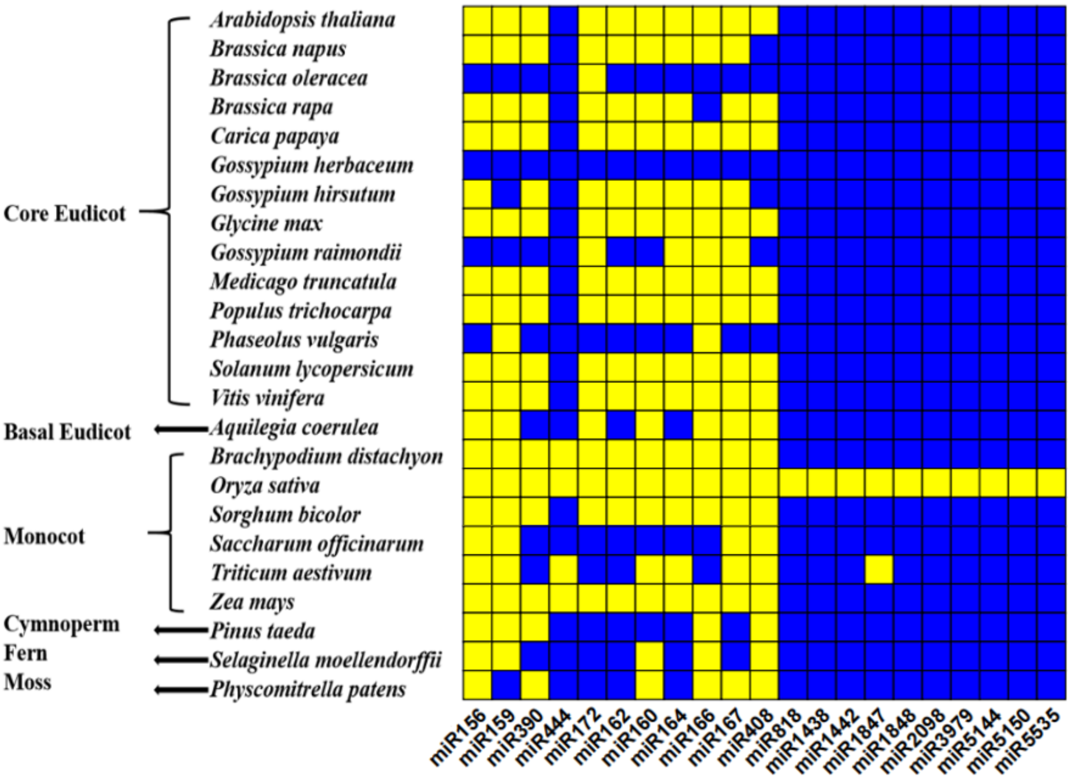

Figure S1. The distribution of miRNAs in plants. We assigned a rice miRNA into the conserved miRNAs if its family members can be found in other plants according to the family annotations of miRBase[1].

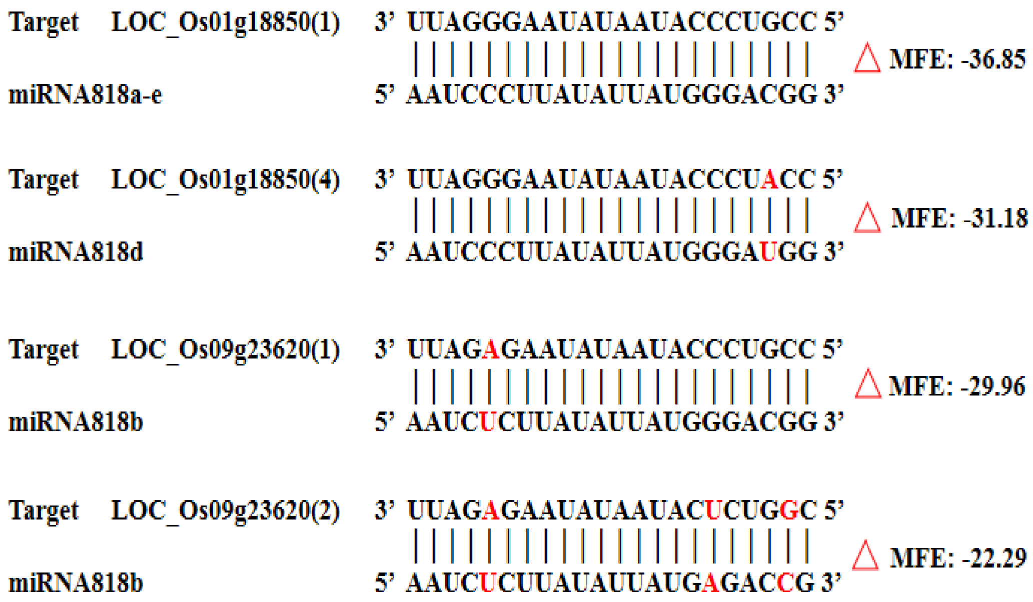

Figure S2. Target genes *LOC_Os01g18850*/*LOC_Os09g23620* targeted by miR818. Mature sequences were extracted from the corresponding pre-miRNAs, base pairing miR818 and their predicted targets are shown with short bars. Sequence mutations compared to the mature sequence in miRBase were marked in red, and the number of these perfect (near perfect)-matching miRNAs are shown in **Table S6**.

Table S1. *Oryza* plant materials used in this study.

No.	Taxa	Group	Origin	Accession Number
1[a,b]	*indica*	Cultivated	Japan	2540
2[a,b]	*indica*	Cultivated	Thailand	5803
3[b]	*indica*	Cultivated	Madagascar	6087
4[b]	*indica*	Cultivated	Bangladesh	6590
5[a,b]	*indica*	Cultivated	India	7919
6[b]	*indica*	Cultivated	Brazil	7937
7	*indica*	Cultivated	Vietnam	8228
8[b]	*indica*	Cultivated	Taiwan	8268
9[b]	*indica*	Cultivated	Sri Lanka	8952
10[b]	*indica*	Cultivated	Philippines	30416
11	*indica*	Cultivated	India	45011
12[b]	*indica*	Cultivated	Nepal	58930
13[b]	*indica*	Cultivated	Myanmar	33131
14[a,b]	*indica*	Cultivated	Taiwan	105
15[b]	*indica*	Cultivated	Indonesia	9147
16[a,b]	*indica*	Cultivated	China	80824
17[a,b]	*O. nivara*	Wild	Myanmar	81941
18[a,b]	*O. nivara*	Wild	Cambodia	106025
19[a,b]	*O. nivara*	Wild	India	105327
20[a,b]	*O. nivara*	Wild	Laos	106151
21[a,b]	*O. nivara*	Wild	Thailand	104440
22[a,b]	*O. rufipogon*	Wild	Thailand	104746
23[a,b]	*O. rufipogon*	Wild	India	81884
24	*O. rufipogon*	Wild	India	105308
25[b]	*O. rufipogon*	Wild	Philippines	80774
26[a,b]	*O. rufipogon*	Wild	Taiwan	101193
27[b]	*O. rufipogon*	Wild	Srilanka	105214
28[b]	*O. rufipogon*	Wild	Malaysia	105491
29[b]	*O. rufipogon*	Wild	Indonesia	105567
30[a,b]	*O. rufipogon*	Wild	Cambodia	105726
31[b]	*O. rufipogon*	Wild	Bangladesh	105902
32[b]	*O. rufipogon*	Wild	Laos	106156
33	*O. rufipogon*	Wild	Papua New Guinea	106274
34	*O. rufipogon*	Wild	Myanmar	106379
35[b]	*O. rufipogon*	Wild	Myanmar	106508
36[b]	*O. rufipogon*	Wild	Australia	86542
37	*O. rufipogon*	Wild	India	80433
38	*O. rufipogon*	Wild	Vietnam	81993

39	*O. rufipogon*	Wild	Vietnam	106411
40[a,b]	*O. rufipogon*	Wild	China	
41[a,b]	*temperate japonica*	Cultivated	Japan	5310
42[a,b]	*temperate japonica*	Cultivated	Taiwan	8241
43	*temperate japonica*	Cultivated	Nepal	27630
44[a,b]	*temperate japonica*	Cultivated	Thailand	27716
45	*temperate japonica*	Cultivated	Myanmar	33888
46[a,b]	*temperate japonica*	Cultivated	India	33984
47[b]	*temperate japonica*	Cultivated	Vietnam	40748
48	*temperate japonica*	Cultivated	Korea	55457
49	*temperate japonica*	Cultivated	Afghanistan	58266
50	*temperate japonica*	Cultivated	China	1107
51	*temperate japonica*	Cultivated	Japan	8191
52[a,b]	*temperate japonica*	Cultivated	China	8194
53	*temperate japonica*	Cultivated	Vietnam	8227
54	*temperate japonica*	Cultivated	China	8264
55	*temperate japonica*	Cultivated	Korea	55471
56	*temperate japonica*	Cultivated	Korea	55530
57[b]	*temperate japonica*	Cultivated	Afghanistan	58286
58[a,b]	*tropical japonica*	Cultivated	Japan	2545
59[b]	*tropical japonica*	Cultivated	Philippines	8244
60	*tropical japonica*	Cultivated	Indonesia	8261
61[a,b]	*tropical japonica*	Cultivated	Thailand	24225
62[b]	*tropical japonica*	Cultivated	Pakistan	38698
63[a,b]	*tropical japonica*	Cultivated	Brazil	50448
64[a,b]	*tropical japonica*	Cultivated	United States	1715
65	*tropical japonica*	Cultivated	Brazil	3297
66	*tropical japonica*	Cultivated	Philippines	3782
67	*tropical japonica*	Cultivated	India	6264
68[b]	*tropical japonica*	Cultivated	Bangladesh	25901
69	*tropical japonica*	Cultivated	Pakistan	27869
70	*tropical japonica*	Cultivated	Bangladesh	29119
71	*tropical japonica*	Cultivated	Indonesia	43325
72	*tropical japonica*	Cultivated	Indonesia	43372
73	*tropical japonica*	Cultivated	Indonesia	43675
74[a,b]	*tropical japonica*	Cultivated	India	44192
75[a,b]	*O. barthii*	Wild	Senegal	101958

[a] Rice materials used for resequencing of target sites for conserved miRNAs

[b] Additional rice materials for *Os03g29680*

Note: all materials listed in the table were used to resequence target sites of rice-specific miRNAs

Table S2. List of predicted target genes involved in flower development in rice.

miRNAs	Target Genes	References [a]	Categories [b]	Family/Annotation	Locations	N[c]	S [d]	
							Wild rice	Cultivated rice
miR156	*LOC_Os01g69830*	[1,2,3]	conserved	SBP	CDS	54	0	0
miR156	*LOC_Os02g04680*	[1,2,3]	conserved	SBP	CDS	54	0	0
miR156	*LOC_Os02g07780*	[1, 2]	conserved	SBP	3'UTR	54	0	0
miR156	*LOC_Os04g46580*	[1,3]	conserved	SBP	CDS	54	0	0
miR156	*LOC_Os06g45310*	[1,2,3]	conserved	SBP	CDS	54	0	0
miR156	*LOC_Os06g49010*	[1,2,3]	conserved	SBP	CDS	54	0	0
miR156	*LOC_Os07g32170*	[1,2,3]	conserved	SBP	3'UTR	54	0	0
miR156	*LOC_Os08g39890*	[1,2,3,4]	conserved	SBP	CDS	54	0	0
miR156	*LOC_Os08g41940*	[1, 3]	conserved	SBP	CDS	54	0	0
miR156	*LOC_Os09g31438*	[1,2,3]	conserved	SBP	CDS	54	0	0
miR156	*LOC_Os09g32944*	[1,2,3]	conserved	SBP	CDS	54	0	0
miR156	*LOC_Os11g30370*	[1,3]	conserved	SBP	CDS	/	/	/
miR159	*LOC_Os01g59660*	[2,3,4,5,6]	conserved	MYB	CDS	54	0	0
miR159	*LOC_Os03g38210*	/	conserved	MYB	CDS	44	0	0
miR159	*LOC_Os05g41166*	/	conserved	MYB	CDS	54	0	0
miR390	*LOC_Os11g36140*	/	conserved	Receptor-like protein kinase 2 precursor	CDS	50	0	0
miR390	*LOC_Os02g10100*	[3,4,7]	conserved	Protein kinase EXS precursor	CDS	54	0	0
miR444	*LOC_Os02g36924*	[2,8]	conserved	MADS	CDS	54	0	0

miR444	*LOC_Os02g49840*	[2,8]	conserved	MADS	CDS	54	0	0
miR529	*LOC_Os01g69830*	[3]	conserved	SBP	CDS	54	0	0
miR529	*LOC_Os08g39890*	[2,3]	conserved	SBP	CDS	54	0	0
miR529	*LOC_Os09g31438*	[3]	conserved	SBP	CDS	54	0	0
miR172	*LOC_Os03g60430*	[3,9,10]	conserved	AP232/EREBP	CDS	54	0	0
miR172	*LOC_Os07g13170*	[3,10]	conserved	AP293/EREBP	CDS	54	0	0
miR172	*LOC_Os06g43220*	[3,10]	conserved	AP287/EREBP	CDS	54	0	0
miR172	*LOC_Os04g55560*	[3,10]	conserved	AP250/EREBP	CDS	46	0	0
miR172	*LOC_Os05g03040*	[3,9,10,11]	conserved	AP254/EREBP	CDS	54	0	0
miR162	*LOC_Os03g02970*	[2,3]	conserved	Endoribonuclease Dicer	CDS	54	0	0
miR160	*LOC_Os06g47150*	[2,3]	conserved	ARF	CDS	/	/	/
miR160	*LOC_Os04g43910*	[2,3]	conserved	ARF	CDS	/	/	/
miR160	*LOC_Os07g29360*	/	conserved	tetratricopeptide repeat domain	CDS	/	/	/
miR164	*LOC_Os06g23650*	[3]	conserved	no apical meristem protein	CDS	/	/	/
miR166	*LOC_Os12g41860*	[2,3]	conserved	START domain containing protein	CDS	/	/	/
miR166	*LOC_Os03g43930*	[2,3,11]	conserved	START domain containing protein	CDS	/	/	/
miR167	*LOC_Os04g57610*	[13]	conserved	ARF8	CDS	/	/	/
miR408	*LOC_Os03g15340*	/	conserved	plastocyanin-like domain	CDS	/	/	/
miR408	*LOC_Os06g15600*	/	conserved	plastocyanin-like domain	CDS	/	/	/
miR408	*LOC_Os03g50140*	[3][14]	conserved	plastocyanin-like domain	CDS	/	/	/
miR408	*LOC_Os02g49850*	/	conserved	plastocyanin-like domain	CDS	/	/	/
	LOC_Os09g35690	/	non-conserved	zinc RING finger protein	3'UTR	/	/	/

miR818	*LOC_Os09g23620*	/	non-conserved	MYB family transcription factor, putative, expressed	3'UTR	152	3	2
miR818	*LOC_Os01g18850*	/	non-conserved	OsSPL1 - SBP-box gene family member	3'UTR	152	4	2
miR818	*LOC_Os11g47160*	/	non-conserved	Receptor kinase 1	CDS	/	/	/
miR1438	*LOC_Os09g35690*	/	non-conserved	zinc RING finger protein	CDS	/	/	/
miR1442	*LOC_Os02g58670*	[3]	non-conserved	bZIP transcription factor	3'UTR	/	/	/
miR1847	*LOC_Os11g31620*	/	non-conserved	OsFBL55 - F-box domain and LRR containing protein	CDS	/	/	/
miR1848	*LOC_Os02g06410*	/	non-conserved	CBS domain containing membrane protein	CDS	/	/	/
	LOC_Os02g10100	/	non-conserved	leucine-rich repeat receptor protein kinase EXS precursor	CDS	/	/	/
miR2098	*LOC_Os08g41940*	/	non-conserved	OsSPL16	5’UTR	/	/	/
miR3979	*LOC_Os06g06750*	/	non-conserved	OsMADS5 MADS-box family	5’UTR	/	/	/
miR5144	*LOC_Os02g12790*	/	non-conserved	GATA zinc finger domain	3'UTR	/	/	/
miR5150	*LOC_Os01g70260*	/	non-conserved	receptor-like protein kinase 2 precursor	3'UTR	/	/	/
miR5535	*LOC_Os06g51260*	/	non-conserved	MYB family transcription factor	CDS-3'UTR	/	/	/

[a] Previously reported references which have confirmed our predicted targets;

[b]We assigned one rice miRNA into the conserved miRNAs if its family members can be found in other species according to the family annotations of miRBase[12];

[c] Number of the analyzed chromosomes;

[d] Number of segregating sites in binding sites;

Note: The number of segregating sites occurring in binding sites is given for the examined target genes in wild and cultivated rice populations.

The overlapping binding sites were indicated in bold, and the slash line showed the target genes which were not amplified.

LITERATURE CITED

[1] Xie, K., Wu, C. and Xiong, L., 2006. Genomic organization, differential expression, and interaction of SQUAMOSA promoter-binding-like transcription factors and microRNA156 in rice. Plant Physiol. 142, 280-93.

[2] Li, Y.-F., et al., 2010. Transcriptome-wide identification of microRNA targets in rice. The Plant Journal 62, 742-759.

[3] Zhou, M., et al., 2010. Degradome sequencing reveals endogenous small RNA targets in rice (*Oryza sativa* L. ssp. *indica*). Frontiers in Biology 5, 67-90.

[4] Guo, X., et al., 2008. Selection and mutation on microRNA target sequences during rice evolution. BMC Genomics 9, 454.

[5] Luo, Y.C., et al., 2006. Rice embryogenic calli express a unique set of microRNAs, suggesting regulatory roles of microRNAs in plant post-embryogenic development. FEBS Lett. 580, 5111-6.

[6] Tsuji, H., et al., 2006. GAMYB controls different sets of genes and is differentially regulated by microRNA in aleurone cells and anthers. The Plant Journal 47, 427-444.

[7] Sunkar, R., Girke, T., Jain, P.K. and Zhu, J.K., 2005. Cloning and characterization of microRNAs from rice. Plant Cell 17, 1397-411.

[8] Lu, C., et al., 2008. Genome-wide analysis for discovery of rice microRNAs reveals natural antisense microRNAs (nat-miRNAs). Proc. Natl. Acad. Sci. U. S. A.

105, 4951-6.
[9] Xue, L.J., Zhang, J.J. and Xue, H.W., 2009. Characterization and expression profiles of miRNAs in rice seeds. Nucleic Acids Res. 37, 916-30.
[10] Zhu, Q.H., Upadhyaya, N.M., Gubler, F. and Helliwell, C.A., 2009. Over-expression of miR172 causes loss of spikelet determinacy and floral organ abnormalities in rice (*Oryza sativa*). BMC Plant Biol. 9, 149.
[11] Liu, Q., et al., 2009. Expression analysis of phytohormone-regulated microRNAs in rice, implying their regulation roles in plant hormone signaling. FEBS Lett. 583, 723-728.
[12] Kozomara, A. & Griffiths-Jones, S. miRBase: integrating microRNA annotation and deep-sequencing data. Nucleic Acids Res. 39, D152-D157 (2011).
[13] Yang JH, Han SJ, Kyung YE, et al. 2006. Evidence of an auxin signal pathway, microRNA167-ARF8-GH3, and its response to exogenous auxin in cultured rice cells. Nucleic Acids Res. 34, 1892-1899.
[14] Zhang JP, Yu Y, Feng YZ, et al. 2017. miRNA MiR408 regulates grain yield and photosynthesis via a phytocyanin protein. Plant Physiology 175, 1175-1185

Table S3. Primer sequences and expected product size for PCR amplifications.

Target Gene	Forward Primer	Reverse Primer	Expected size (bp)
LOC_Os01g18850	GAGGTATGAGAGGTCCTTTCCG	CATTCTACTCCCTCCTTCCCA	593
LOC_Os01g59660	TGAAGCCAACTCTACCAGCAAG	GAGGAATGGACCAGGATGTTG	454
LOC_Os01g69830	CACTTCCCTTTCCTGACGG	AAAGGAAGAGCCTGGATTGC	470
LOC_Os02g04680	CGTGGAAAACCTGGGCTCA	GTGGGAGTTGTCATAGGAAGGC	484
LOC_Os02g07780	GCAGCCACTATTTGGATGTTCT	AACACAAGCAAGCCATCACTC	624
LOC_Os02g10100	ATCTGAGGACATTCCAATACAG	CCGATAAGTCCAGCATTGTGA	533
LOC_Os02g36924	TCCGTATTATCTCATGCCACAA	TATTTGGGTTGATTGGTAGGTG	381
LOC_Os02g49840	CGTATGTGATTGAGTTGTGTGTGA	CTCACCCATCAGTTGCCTACA	621
LOC_Os03g02970	TGGGTGCCTTCACAGATACGC	CGTGCCCTCTGAAGTGGATTG	877
LOC_Os03g38210	TCAAGAACTACTGGAACACGAGGAC	GAGTGATCTGTGAGTCGAGGTAAATG	1046
LOC_Os03g60430	AGTTGTTAGATACCGTTTCCCTTGTG	ATGGAGAAGACAGAGAGATCAATGA	879
LOC_Os04g46580	GTTATTAGGACTGATGGTGTGC	CTGTCGGTCGGAGAAGAAT	321
LOC_Os04g55560	CATTCAGTCCCAGGCATCATCAC	TGTTCCACATCTCGGCAGTTACG	1138
LOC_Os05g03040	GTTAGACTGGTTTGCCGTTAGACA	AGGTGTGTGGATCAATACATCTCAG	870
LOC_Os05g41166	CGTTCGCCTTTGTATCCTG	GCTCTTCCGACGATACTGG	764
LOC_Os06g43220	ATTGCTAACCACGGGCTACAGGA	ACGGGTCGTCCACGGTACAAATA	688
LOC_Os06g45310	AGCAATCCAGCATTCCAGTTA	TTAGAGTTTCCTTCGCTTGGTA	495
LOC_Os06g49010	GAATACACCAGGCACAAGCAAC	CAACCCGTTCCAAATAGATGC	639
LOC_Os07g13170	CGGGCTTCTTTGTGAACCTCAG	ACCTGGTAGCACGGTCACGATT	1162

LOC_Os07 g32170	ATGCTATAGCTTTCGGT GGTT	GGGCAAAGGCAATAGT ACAAT	815
LOC_Os08 g39890	GGGTTCCAAGCAGCGT AA	CATCGTGTTGCTGGTTT GG	611
LOC_Os08 g41940	GCTGGACAGGGATGAT CAAA	ACTTGGCACCATCATGT TGAG	931
LOC_Os09 g23620	ATGGTGGCGAGAGATG AGACTA	CTCTCTCTCACACAACC ACTTCAC	865
LOC_Os09 g31438	GCAGTTGCGGGATACA GTG	AGTGACGGTGGGGAAG AAG	564
LOC_Os09 g32944	GTGGGCATGGTGAGTTA ATCA	GCCGTGGTAGTTCATCT CGT	986
LOC_Os11 g36140	CACTAATGCTCTCCAAG GCACTCT	CAGTGCTTGGTATTTCA CCGCTA	1073

Note：The target sites for miR529b overlap with the ones for miR156, and they share a pair of primers.

Table S4. Primer pairs for amplification of pre-miRNAs.

No.	miRNA	Primer sequence	Expected length (bp)
1	miR818a	F-CCGTCCAAAAGACCCAACCT	474
		R-AGTACTTTAGGCACAACTTGTCA	
2	miR818b	1. F-TTGGTGACCGACGACCAAAT	347
		R-TTGCGATGGCAACGATGATG	
		2. F- GCTCACCTAGCTCATCCACG	900
		R- ATACCGTTCGGCTTCCACAA	
3	miR818c	F-CCTGGATTCACCAGGACGAC	473
		R-CGCTTTGAGGGCGATTTCTG	
4	miR818d	F-GCCAACTAGCAATGGTCCCT	493
		R-AGTCTCTGAAACCAGCAGCC	
5	miR818e	1. F-TGCGCGTGATGTAGGGTTTA	616
		R-TCAAACGTTGGCACGGAAAC	
		2. F-TGCGCGTGATGTAGGGTTTA	885
		R-TCTGAAGCTCCCCTTTTGGG	

Table S5. Average diversity measures of miRNAs involved in flower development in rice cultivars and wild populations.

Populations	Regions	π (per bp)	SNP density (SNPs/bp)
**Cultivated rice**	**5' flanking regions**	0.00433±0.00089	0.00605±0.00121
	**Binding sites**	0.00038±0.00028	0.00021±0.00016
	**3' flanking regions**	0.00397±0.00076	0.00405±0.00069
**Wild rice**	**5' flanking regions**	0.00980±0.00348	0.00808±0.00249
	**Binding sites**	0.00322±0.00281	0.00302±0.00203
	**3' flanking regions**	0.00934±0.00245	0.00722±0.00166

Note: The values represent the standard errors of the mean values (mean ± SE).

Table S6. The genetic variation of the two rice-specific microRNA sequences.

miRNAs	*N**	Mature sequence#	Putative miRNA : Target pairing
**miR818a**	15		
	9	AATCCCTTATATTATGGGACGG	*LOC_Os01g18850*type1/2/4/5 *LOC_Os09g23620*type1/2
	3	AATCCCTTATA**C**TATGGGA**T**GG	*LOC_Os01g18850*type1/4
	1	AATCCCTTATA**C**TATGGGA**TA**G	*LOC_Os01g18850*type2/4
	1	AATCCCTTATATTAT**A**GGACGG	*LOC_Os01g18850*type2/4 *LOC_Os09g23620*type1
	1	AATCCCTTATATTA**G**GGGACGG	*LOC_Os01g18850*type1/2/4/5 *LOC_Os09g23620*type1
**miR818b**	15		
	11	AATCCCTTATATTATGGGACGG	*LOC_Os01g18850*type1/2/4/5 *LOC_Os09g23620*type1/2
	1	AATCCCTTATATTATG**AA**ACGG	*LOC_Os01g18850*type5; *LOC_Os09g23620*type1/2
	1	AATC**T**CTTATATTATGGGACGG	*LOC_Os01g18850*type1/2/3/4; *LOC_Os09g23620*type1/2
	1	AATCCCTTATATTATGG**A**ACGG	*LOC_Os01g18850*type1/2/4 ; *LOC_Os09g23620*type1
**miR818c**	15		
	6	AATCCCTTATATTATGGGACGG	*LOC_Os01g18850*type1/2/4/5 *LOC_Os09g23620*type1/2
	4	AAT**T**CCTTATATTATGGGA**T**G**A**	*LOC_Os01g18850*type1/3/4/5
	2	AATCCCTTATATTAT**A**GGACG**A**	*LOC_Os01g18850*type1/4
	2	AATCCCTTATATTAT**AA**GACG**A**	*LOC_Os01g18850*type5
	1	AATCCCTTATATTATGG**A**ACGG	*LOC_Os01g18850*type1/2/4 ; *LOC_Os09g23620*type1
**miR818d**	15		
	3	AATCCCTTATATTATGGGACGG	*LOC_Os01g18850*type1/2/4/5 *LOC_Os09g23620*type1/2
	11	AATC**A**CTTATATTATGGGACGG	*LOC_Os01g18850*type1/2/3/4; *LOC_Os09g23620*type1/2
	1	AATCCCTTATATTATGGGA**T**GG	*LOC_Os01g18850*type1/4/5; *LOC_Os09g23620*type1
**miR818e**	15		
	7	AATCCCTTATATTATGGGACGG	*LOC_Os01g18850*type1/2/4/5 *LOC_Os09g23620*type1/2

	6	AATCCCTTATATTAT**A**GGACGG	*LOC_Os01g18850*type2/4; *LOC_Os09g23620*type1
	1	AATC**T**CTTATATTAT**A**GGACGG	*LOC_Os01g18850*type1/2/4; *LOC_Os09g23620*type1
	1	AATCCCT**AT**TATTAT**A**GGACGG	
